# Fungi as mutualistic partners in ant-plant interactions

**DOI:** 10.3389/ffunb.2023.1213997

**Published:** 2023-10-02

**Authors:** Veronika E. Mayer, Hermann Voglmayr, Rumsais Blatrix, Jérôme Orivel, Céline Leroy

**Affiliations:** ^1^ Department of Botany and Biodiversity Research – Division of Structural and Functional Botany, University of Vienna, Wien, Austria; ^2^ Department of Botany and Biodiversity Research – Mycology Research Group, University of Vienna, Wien, Austria; ^3^ CEFE, University of Montpellier, CNRS, EPHE, IRD, Montpellier, France; ^4^ EcoFoG, AgroParisTech, CIRAD, CNRS, INRAE, Université des Antilles, Université de Guyane, Kourou, France; ^5^ AMAP, Université de Montpellier, CIRAD, CNRS, INRAE, IRD, Montpellier, France

**Keywords:** ants, Chaetothyriales, Capnodiales, specificity, transmission, evolutionary history, function

## Abstract

Associations between fungi and ants living in mutualistic relationship with plants (“plant-ants”) have been known for a long time. However, only in recent years has the mutualistic nature, frequency, and geographical extent of associations between tropical arboreal ants with fungi of the ascomycete order Chaetothyriales and Capnodiales (belonging to the so-called “Black Fungi”) become clear. Two groups of arboreal ants displaying different nesting strategies are associated with ascomycete fungi: carton-building ants that construct nest walls and galleries on stems, branches or below leaves which are overgrown by fungal hyphae, and plant-ants that make their nests inside living plants (myrmecophytes) in plant provided cavities (domatia) where ants cultivate fungi in small delimited “patches”. In this review we summarize the current knowledge about these unsuspected plant-ant-fungus interactions. The data suggest, that at least some of these ant-associated fungi seem to have coevolved with ants over a long period of time and have developed specific adaptations to this lifestyle.

## 1 Introduction

Associations between fungi and insects are manifold, ranging from pathogenic (Hajek and Leger, 1994) to mutualistic (Biedermann and Vega, 2020). The most well-known mutualistic association in ants is the nutritional relationship between fungus growing ants (tribe Attini, subtribe Attina) and basidiomycete fungi (Agaricales: Agaricaceae and Pterulaceae) which are usually cultivated as mycelia and used as the primary food source for the ant colony (Weber, 1972; Schultz and Brady, 2008). However, there are other less well-known mutualistic interactions between ants and ascomycete fungi identified as Chaetothyriales, with Capnodiales also occurring frequently (Defossez et al., 2009; Mayer and Voglmayr, 2009; Ruiz-Gonzalez et al., 2011; Voglmayr et al., 2011; Nepel et al., 2014; Kokolo et al., 2016; Vasse et al., 2017; Gegenbauer et al., 2023). Both Chaetothyriales and Capnodiales belong to the so-called “Black Fungi”, a diverse group of slow-growing ascomycetes with melanized hyphae. Black Fungi have been found in mutualistic association with Old World *Lasius* ants on the walls of nests inside dead trees (Lagerheim, 1900; Maschwitz and Hölldobler, 1970; Schlick-Steiner et al., 2008), with mound-building *Formica* ants (Lindström et al., 2018; Lindström et al., 2021), and with various arboreal ants in the tropics. The latter association between arboreal ants and fungi is the focus of the present review because this type of association has received much attention within the past 15 years.

Arboreal ants (= species that forage and nest mostly or entirely on plants) make up about a third of the total ant fauna in tropical rainforests (Longino and Colwell, 2020). A time-scaled ant phylogeny suggests that arboreal foraging evolved in the early Cretaceous followed by arboreal nesting in the late Cretaceous (Nelsen et al., 2018). Under conditions of limited nesting space availability in arboreal ant communities in tropical forests (Wilson, 1987; Fiala and Maschwitz, 1992; Fonseca, 1993; Camarota et al., 2020) that accompanies the high diversity of ants (Kass et al., 2022; Schultheiss et al., 2022) niche differentiation by development of different nesting strategies may be a crucial factor for arboreal ant communities to allow a broader use of limited resources.

**Figure 1:**
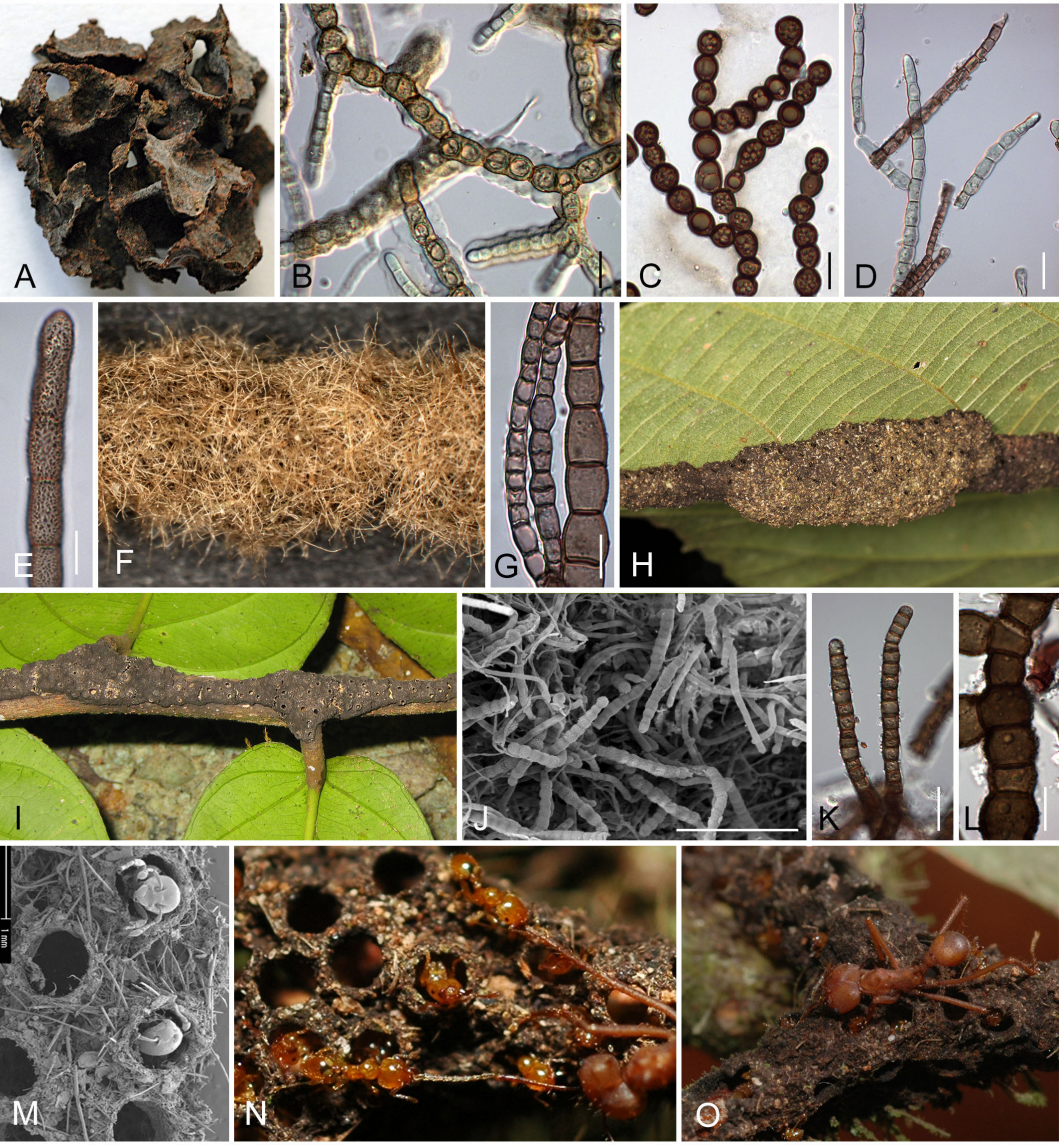
Nest walls, galleries and carton in arboreal ant nests with fungi from “Functional group 1”. **(A–C)** Fungi from Capnodiales, **(D–O)** fungi from Chaetothyriales. **(A, B)** *Crematogaster* ant carton nest from Cameroon (sample BN-Lon33), **(B)** Capnodiales hyphae from this carton sample germinating on 2% MEA. **(C)** Capnodiales hyphae with dark brown globose monilioid cells from a nest in Malaysia inhabited parabiotically by *Crematogaster* sp. and *Camponotus* sp. (sample M-Camp2). **(D)** Chaetothyriales hyphae germinating on 2% MEA from a *Crematogaster* sp. ant carton (sample CN-Cre-BO3) and **(E)** elongated monilioid cells with coarsely verrucose walls of a *Pheidole* carton nest (sample CN-Phe1), both from Cameroon. **(F)** *Monomorium* ant nest from Malaysia (sample M-Mo) built of loosely interwoven plant trichomes stabilized with Chaetothyriales hyphae. **(G)** Hyphae of two Chaetothyriales strains from the *Monomorium* ant nest shown in **(F)**. **(H)** Nest of *Technomyrmex* sp. found in Borneo on a lower leaf surface and stabilized with hyphae. **(I)** Dark-brown runway gallery of *Azteca brevis* on a branch of *Tetrathylacium macrophyllum* in Costa Rica. **(J)** The scanning electron microscope picture shows that the carton of the galleries consists of plant material with densely intertwined chaetothyrialean hyphae. **(K, L)** Strains with different morphology were found. **(M)** Carton gallery of *Allomerus decemarticulatus* on *Hirtella physophora* from French Guiana built from plant trichomes which the ants obtained by clearing a path and stabilized with chaetothyrialean hyphae. During the construction process, holes are left which are guarded by *Allomerus* workers throughout the day. **(N, O)** The construction and stability of the galleries built by *Allomerus* ants allow a peculiar ambush tactic to capture prey. **(N)** *Allomerus* workers had taken up positions beneath the holes with their mandibles wide open grasping the legs or antennae of other arthropods. **(O)** The prey is immobilised by stretching its legs, antennae or wings against the gallery so that the workers can kill it. Bars, **(E, G, K, L)** 10 µm, **(B–D)** 20 µm, **(J)** 100µm, **(A)** 3mm, **(I, F)** 5mm, **(M)** 1mm. **(H)** with courtesy of F. Etl. MEA, malt extract agar.

**Figure 2:**
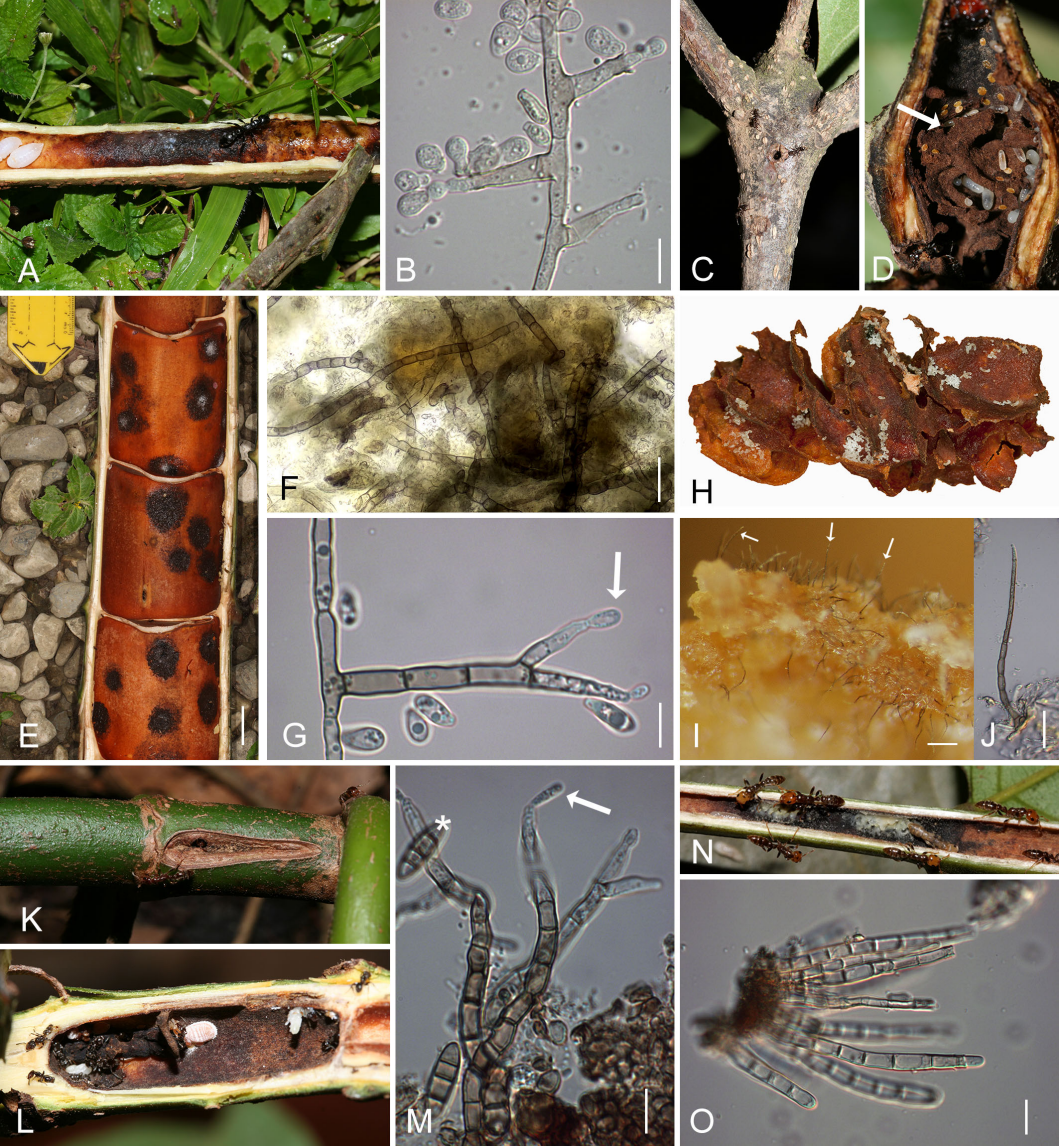
Domatia of ant-plants (myrmecophytes) with patches that contain chaetothyrialean fungi. **(A)** Opened branch of *Barteria fistulosa* (Passifloraceae) from Cameroon with *Tetraponera aethiops* and a black patch with Chaetothyrialeas. **(B)** The fungal symbiont (CBS 134920) of *T. aethiops* growing on 2% MEA showing conidiophores with conidia. **(C, D)** *Cordia alliodora* (Boraginaceae) from Costa Rica with swollen hollow nodes which are inhabited by *Azteca* sp. **(D)** The domatium cavity is structured into compartments with a carton (arrow). **(E)** Opened *Cecropia obtusifolia* stem inhabited with *Azteca constructor*. The black round patches are containing Chaetothyriales. **(F)** A squash mount of fresh patches from *C obtusifolia* inhabited by *A. constructor* made in the field. Numerous hyphae (stained with calcofluor white) pervade the organic matter (blurry parts) of the patches. **(G)** Hyphae of a pure culture (CBS 132003) of the *Azteca/Cecropia* association growing on 2% MEA with spores and conidiophorous cells (arrow). **(H-J)** Carton made of scratched parenchyma tissue from the inner domatium wall **(H)** of the same *Cecropia* individual as in **(E)** with eggs and larvae (white dots) on the carton surface. **(I)** Numerous needle-like black conidiophores (arrows) were found on this carton. **(J)** Close-up of a needle like conidiophore from **(I)**. **(K)** *Triplaris americana* (Polygonaceae) branch with an entrance hole from outside, **(L)** opened showing a domatium inhabited with *Pseudomyrmex* sp. and a black fungal patch. **(M)** Conidiophores (arrow) with conidia (asterisk) from a patch in a *Triplaris americana* domatium. **(N)** Opened leaf petiole of *Tachigali paniculata* inhabited by *Pseudomyrmex penetrator* ants, showing blackish fungal patches in some places covered with masses of nematodes (white parts). **(O)** Conidiophores from the patch shown in **(N)**. Bars, **(A, E, K–M)** 2cm, **(C, D, H)** 1cm, **(B, G, I, M)** 10µm, **(F, O)** 20µm. MEA, maltose extract agar.

**Figure 3:**
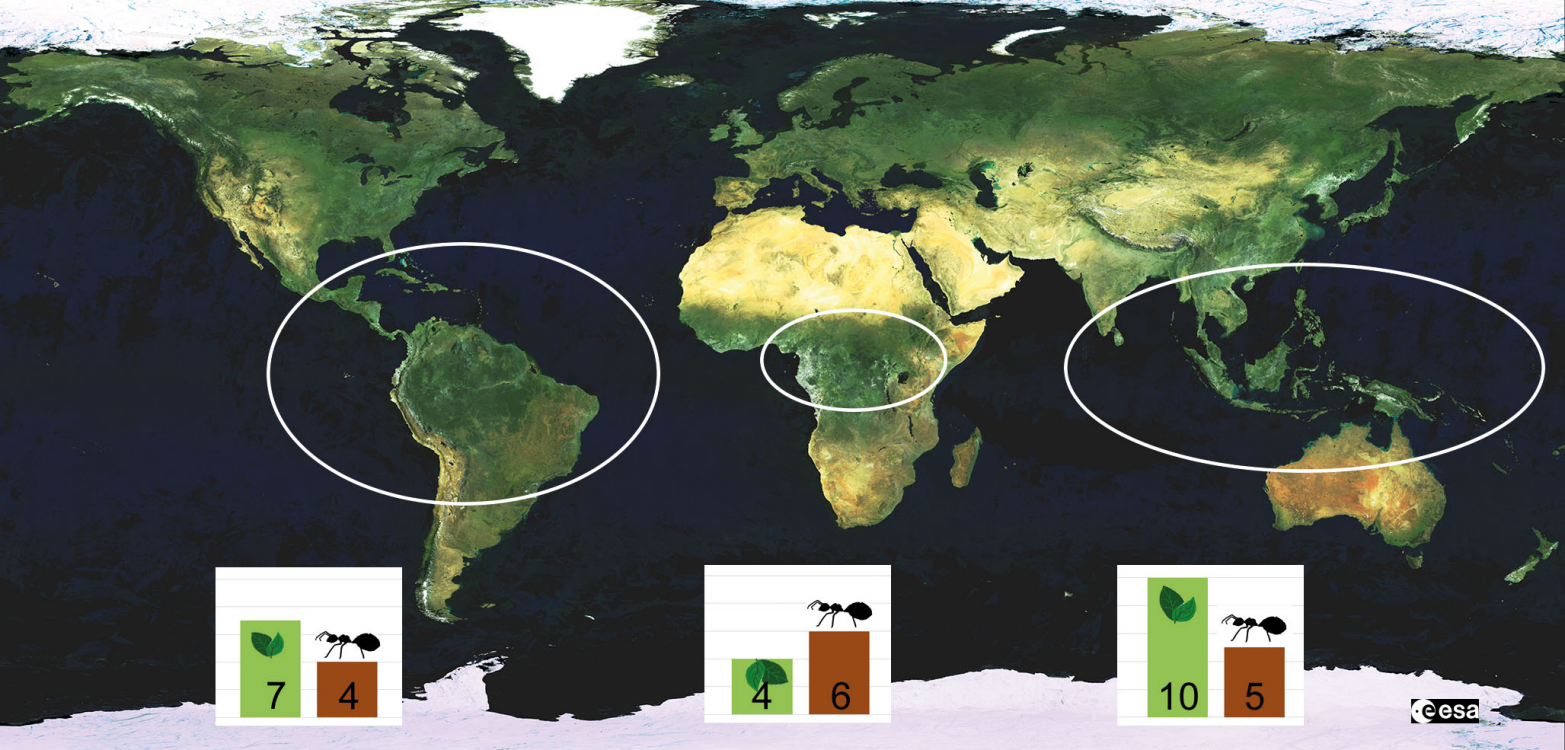
Geographical distribution of Chaetothyriales growing in ant nests in hollow plant structures (domatia). The bars indicate the number of plant (green) and ant (brown) genera that are known to house ant-associated fungi. The numbers are based on Table 1 in Mayer et al., 2014. While fungi growing in domatia are restricted to the tropics, carton fungi can also be found in temperate regions.

Three categories of arboreal ants differing in their nesting strategy can be defined: (1) a group of opportunists that use a range of cavities and crevices in plants as incidental nest sites to shelter the colonies (Hölldobler and Wilson, 1990; Novais et al., 2017; Philpott et al., 2018), many of them making constructions with fibers, resin, soil, sand, and organic matter to reduce entrance sizes and improve the cavity defense (Priest et al., 2021). (2) a group of carton-builders that make *de novo* constructions of carton nests and/or galleries independent from existing cavities; construction materials are plant derived bark and wood fragments, trichomes, mosses and epiphylls, and hyphae of ascomycete so-called “Black Fungi” (Weißflog, 2001; Mayer and Voglmayr, 2009; Ruiz-Gonzalez et al., 2011). Ant-constructed carton with fungi can constitute walls of free-hanging nests on the lower surface of leaves as in *Crematogaster* and *Technomyrmex* (Weißflog, 2001; Voglmayr et al., 2011) (see Figure 19 in Weißflog, 2001) (**Figure 1H**), runway galleries along stems, branches and leaf surfaces as found on *Tetrathylacium macrophyllum* built by *Azteca brevis* ants (Mayer and Voglmayr, 2009; Mayer et al., 2017) (**Figures 1I, O**), or cardboard-like “carton” structures sheathing entrances to their nest side, protecting flocks of scale insects, or partitioning the nest interior (**Figure 1A**). Interwoven plant hairs manured with the excrements of the ants like in *Monomorium* sp. (Weißflog, 2001) (**Figure 1F**), or in the *Hirtella physophora* - *Allomerus decemarticulatus* association (Dejean et al., 2005) (**Figure 1M**) are also used. (3) a category of plant-ants that live inside “myrmecophytes”, plants with organs that have been transformed into hollow cavities called “domatia” which can be derived from stems (**Figures 2A, C-E, K, L, N**), leaf pouches, leaf petioles, stipules, root tubers, rhizomes, or hypocotyls (Chomicki and Renner, 2015), being induced by the plants themselves without any induction from plant-ants (but see Blüthgen and Wesenberg, 2001; Gaume et al., 2005). In addition to shelter, some myrmecophytes offer an immediate source of nutrients for the inhabiting ant colony through carbohydrate-rich extrafloral nectaries (Heil et al., 2000; Blüthgen et al., 2004; Gonzalez-Teuber and Heil, 2009) and, in some cases, protein- and lipid-rich food bodies (Folgarait and Davidson, 1994, Folgarait and Davidson, 1995; Heil et al., 1998; Fischer et al., 2002; Hatada et al., 2002; Heil et al., 2004; Webber et al., 2007; Bischof et al., 2013). Myrmecophytes are found almost exclusively in the tropical regions of Australasia, Africa and the New World (Chomicki and Renner, 2015), where the pressure of pathogens and herbivores is high, and nutrients are limited (Davidson, 1997; Kaspari and Yanoviak, 2001). Like in free nest structures, Black Fungi are also present in domatia (**Figure 2**). This has been documented for a long time (Miehe, 1911; Bailey, 1920), but due to the unspectacular appearance, difficulties in cultivation and taxonomic assignment, and lack of conclusive evidence of mutualism between fungi and the myrmecophytic partners, fungi in ant-plant associations did not receive much attention. Only recently, the development of molecular techniques has allowed the taxonomic assignment of fungi found in many unrelated ant-plant associations in the tropics worldwide (**Figure 3**).

## 2 Two functional groups of Black Fungi

**Figure 4:**
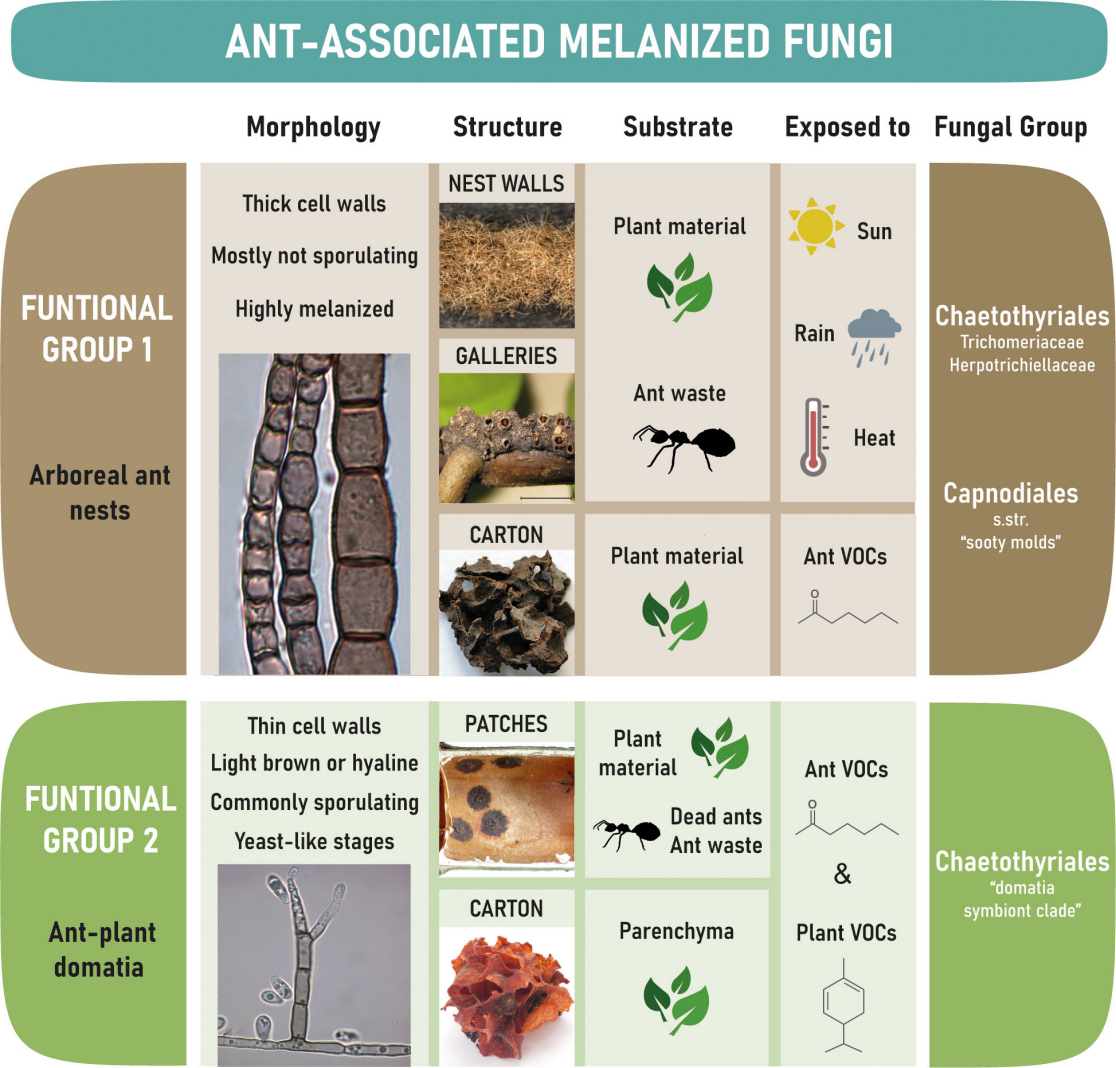
Overview on the two major groups of tropical ant-associated melanized fungi, the substrate and environmental conditions they are exposed to.

Black Fungi associated with ants can be subdivided into two main functional groups that differ substantially: group 1 are those that occur on ant-constructed free-hanging walls of nests and galleries built by the ants, whilst group 2 are those that occur inside the domatia of myrmecophytes (**Figure 4**).

### 2.1 Functional Group 1: exposed to the environment on nest walls and galleries (“carton-fungi”)

Ant-associated fungi of Functional Group I are exposed to the environment and must be able to withstand extreme conditions like heavy rain, high temperatures, desiccation, intense solar radiation, nutrient scarcity, and a constant exposure to fungal competitors. Chaetothyriales (Ascomycota, Eurotiomycetes) and Capnodiales (including Mycosphaerellales) (Ascomycota, Dothideomycetes) were frequently isolated from hyphal fragments of nest walls, galleries, and carton structures, as were Venturiaceae (Ascomycota, Dothideomycetes, Pleosporales) in rare cases (Mayer and Voglmayr, 2009; Voglmayr et al., 2011; Seipke et al., 2012; Nepel et al., 2014; Dejean et al., 2023). Strains that occur in this environment have highly melanized cell walls (**Figures 1B-E, G, K, L**) as melanization is enabling fungi to tolerate physical stress (Cordero and Casadevall, 2017). Fungi of this group rarely sporulate (Voglmayr et al., 2011), which is common for Trichomeriaceae (Ascomycota, Eurotiomycetes, Chaetothyriales) in the natural habitat (Chomnunti et al., 2012; Quan et al., 2022); instead they may be dispersed primarily as hyphal fragments by the ants or by air and water movement.

Genomes of two nest-wall species from Chaetothyriales did not show particular differences compared to species not associated with ants (Teixeira et al., 2017; Quan et al., 2022) and do not seem to have evolved specific adaptations.

### 2.2 Functional Group 2: hidden inside myrmecophytes (“domatia fungi”)

Black Fungi of the second functional group live within the domatia of myrmecophytes and, therefore, in a completely distinct micro-habitat to the first group. Hot-spots for fungal growth are round, blackish “patches” of a very regular shape and thickness (**Figures 2A, E, L, N**) which are actively constructed by the ants by piling up parenchyma scratched from the respective domatia walls, or, in older domatia, by depositing exoskeletons of dead nest members (Blatrix et al., 2009; Defossez et al., 2009; Nepel et al., 2016; Mayer et al., 2018). Thus, the activities of ants probably have a strong impact on fungal morphogenesis. Apart from patches, fungi may grow on structures built by some ant species from chewed plant material to divide the domatium space into compartments (e.g. in the *Cordia alliodora – Azteca* sp. association, **Figure 2D**) and to store eggs, larvae, and pupae (e.g. in the *Cecropia obtusifolia* – *Azteca constructor* association, **Figures 2H, J**).

The vast majority of ant-associated fungi in domatia belong to Chaetothyriales (Defossez et al., 2009; Voglmayr et al., 2011; Kokolo et al., 2016; Nepel et al., 2016; Vasse et al., 2017; Lucas et al., 2019; Blatrix et al., 2021; Greenfield et al., 2021). They are characterized by a reduction in cell wall thickness (**Figures 2B, G, M, O**) compared to carton-associated fungi and other Chaetothyriales (Voglmayr et al., 2011). There may be little selective pressure on cell wall thickness and melanization within domatia because of reduced exposure to physical stress, particularly drought, heavy rain and high radiation. This is supported by genome analyses of four domatia-associated chaetothyrialean fungi showing that genes coding for enzymes of the cytochrome P450 family (CYPs), membrane transporters, and alcohol dehydrogenases — thought to be crucial for survival in extreme and hostile climate conditions — revealed contractions in domatia fungi (Moreno et al., 2019) where the sunlight, temperature and drought are less extreme, but not in the fungi growing on the walls of ant nests (Quan et al., 2022) where the fungi are exposed to the extreme environmental conditions. In addition, the role of domatia-associated species in the interaction with ants appears to be partly nutritional (Blatrix et al., 2012a), rather than structural (Dejean et al., 2005; Ruiz-Gonzalez et al., 2011; Mayer et al., 2017) as with carton-associated species (see section 2.1 above). This suggests that ants may have exerted selective pressure for softer hyphae. Another difference to carton-associated strains are the numerous spike-like dark conidiophores (**Figures 2G, I, J, M, O**) and frequent sporulation (Voglmayr et al., 2011).

The genomes of the domatia-associated fungi are remarkably small compared to those of the nest-wall strains (Quan et al., 2022) and other Chaetothyriales (Teixeira et al., 2017), suggesting that at least some domatia-associated species are specialized for this lifestyle.

## 3 What traits predispose Chaetothyriales and Capnodiales to form such frequent associations with ants?

Character state analyses based on phylogenies indicate that the ancestral Chaetothyriales were slow-growing colonizers of rock surfaces characterized by extreme oligotrophic conditions, and in these ancestral ecological niches, they had to cope with co-occurring toxin-producing lichens and cyanobacteria (Gueidan et al., 2008; Quan et al., 2020). Therefore, extremotolerance (temperature, drought, poor nutrient availability) and toxin resistance are crucial ancestral evolutionary adaptations that subsequently opened a window of opportunity to colonize a surprisingly diverse range of extreme habitats that are unable to support the growth of most other potential competitors (Quan et al., 2020). As a result, Chaetothyriales may be predisposed to interact with ants because of their tolerance for various toxic chemical compounds (Teixeira et al., 2017) that are produced by ants for communication (Jackson and Morgan, 1993) and to control diseases (Yek and Mueller, 2011). The production of antibiotic and antimycotic substances (e.g. formic and acetic acid, Tragust et al., 2013) are vital for social insects to ensure health and survival of the colony (Penick et al., 2018), which in turn requires that associated fungi are resistant to these substances as a pre-requisite to inhabit this niche and to establish mutualisms. It therefore makes sense that Chaetothyriales might produce bioactive substances against competing fungi (El-Elimat et al., 2013). In addition, Chaetothyriales appear to be pre-adapted to manipulation by specialized ants, as they thrive under constant and frequent mechanical disturbance due to their pronounced ability of regeneration. The combination of these factors may account for the close relationship between ants and those fungi.

Much less is known about the evolutionary and genomic background of ant-associated Capnodiales. However, they seem to occupy somewhat different niches than the Chaetothyriales, as in different ant-plant fungal systems either the Chaetothyriales or the Capnodiales are dominant. The Capnodiales *sensu lato* represent a morphologically and ecologically highly diverse and speciose lineage with different lifestyles and modes of nutrition, viz. saprobes, plant and human pathogens, mycoparasites, rock-inhabiting fungi, lichenized, epi-, ecto- and endophytes (Abdollahzadeh et al., 2020). Several families of the former Capnodiales *sensu lato* were recently elevated to distinct orders (Abdollahzadeh et al., 2020). As a result, Capnodiales *sensu stricto* were restricted to the sooty molds which colonize plant surfaces covered by sugar-rich honeydew excreted by sap-sucking hemiptera (Abdollahzadeh et al., 2020). Voglmayr et al. (2011) observed that Capnodiales were prominent particularly in “carton” of *Crematogaster* ants, which differed markedly from other ant nests by extremely hard, dense and tough texture, with the fungi only growing on the carton surface. Considering the close association of ants with hemiptera which are tended by the ants in return for carbohydrates, honeydew-inhabiting Capnodiales may be predestined to colonize suitable surfaces within ant nests, if carbohydrates are accidentally or intentionally deposited on them. However, additional investigations are needed to ascertain the nutritional requirements of these Capnodiales isolates.

## 4 From plant surface to ant nests? Evolution of ant-associated Chaetothyriales

As phylogenetic analyses of ant-associated Capnodiales are not available to date, only Chaetothyriales are considered here. Interestingly, the phylogenetic analyses suggest that the strains of the two functional groups exhibit distinct evolutionary patterns. Within Chaetothyriales, species associated with nest-walls and galleries (functional group 1) appear more scattered in the phylogeny compared to species associated with ants living in domatia (functional group 2) (Voglmayr et al., 2011; Nepel et al., 2014; Vasse et al., 2017; Quan et al., 2020). Chaetothyriales of the first group belong to Trichomeriaceae, Herpotrichiellaceae and Cyphellophoraceae and are particularly well represented in the families Trichomeriaceae and Herpotrichiellaceae. This pattern suggests that life as mutualists of ant-made carton evolved multiple times independently, even across different orders. It is not clear whether these species are ant specialists, or if they are opportunistic fungi that primarily live in other environments but are specifically recruited by the ants, or if at least some of them are commensals that arrived without any relation to ants.

In contrast, most domatia-associated Chaetothyriales cluster close to Cyphellophoraceae and Paracladophialophoraceae (Quan et al., 2023) in a monophyletic clade that is composed of domatia-associated species only (“domatia symbiont clade”) and that could potentially constitute a distinct family (Voglmayr et al., 2011; Vasse et al., 2017; Quan et al., 2020). The substantial genome (and gene) reduction reported for species in the domatia symbiont clade (Moreno et al., 2019) suggests a long evolutionary specialization. However, there are also several species of domatia-associated Chaetothyriales that do not belong to the monophyletic main clade but are scattered throughout the phylogeny (Vasse et al., 2017). This may indicate that the ant-associated lifestyle evolved several times. Alternatively, the association with mutualistic ant-plant interactions may be an optional, opportunistic strategy. One example of the latter is *Exophiala oligosperma*, which is known to be a human pathogen found in low-nutrient substrates (De Hoog et al., 2003), and has recently been discovered in ant-occupied domatia of various ant-plants in South-East Asia, and Central and South America (Vasse et al., 2017; Blatrix et al., 2021). In addition, several ant-plant mutualisms on different continents have been found to share a single species of Chaetothyriales, showing the ability of such species to change hosts and continents (Nepel et al., 2016; Vasse et al., 2017; Blatrix et al., 2021; Greenfield et al., 2021). In summary, the evolutionary pattern of domatia-associated Chaetothyriales seems to involve one main radiation possibly linked to specialization on the one hand, and opportunistic nonspecific associations on the other hand. The opportunistic strains isolated from the ant nest environment may not all be involved in interaction with ants.

The phylogenetic patterns of ant-plants and plant-ants are markedly different, as myrmecophytism (i.e. bearing domatia) in plants is distributed throughout the plant phylogeny, with at least 158 independent evolutionary origins, each occurring less than 20 Mya ago (Chomicki and Renner, 2015). In ants the obligate association with myrmecophytes has evolved many times independently, but with three times fewer origins than in the ant-plants (Chomicki and Renner, 2015). Co-diversification between plants and ants seems not to be a driver in the evolution of ant-plant mutualisms. According to the latest phylogenetic reconstruction, the monophyletic clade containing most of the domatia-inhabiting Chaetothyriales evolved at least 27 Mya ago (Quan et al., 2020), that is, earlier than any of the extant lineages of myrmecophytes (Chomicki and Renner, 2015). Two non-exclusive hypotheses, outlined in the following two paragraphs, are proposed to explain how the contemporary associations might have developed despite the distinct evolutionary history of domatia fungi and myrmecophytic plant lineages.

The first hypothesis suggests, that myrmecophytism may have existed much earlier than the recent plant lineages. The development of a domatium is most likely an evolutionarily labile trait that is easily acquired and lost for the following reasons: (i) it has been independently acquired at least 158 times and lost at least 43 times (Chomicki and Renner, 2015), (ii) there are no known plant fossils with domatia, and (iii) in some species, this trait is highly variable (Michelangeli, 2005; Shenoy and Borges, 2010;, Kokolo et al., 2020). Consequently, although the extant lineages of myrmecophytes are less than 20 Mya old, myrmecophytism may have existed much earlier but without leaving any detectable evidence. If a more than 27 Mya old specialized clade of domatia-associated Chaetothyriales exists, as suggested by the most recent dated phylogeny (Quan et al., 2020), it could have originated from myrmecophytic plant lineages that are now extinct. The adaptation to ant-inhabited domatia as habitat could have been maintained through host-switching or opportunistic life style outside domatia, both of which are strategies that do not seem to be rare in extant species (see previous paragraphs).

The second hypothesis suggests, that fungi associated with ants may have originated from epiphyllous species. In the current state of knowledge, most ant-associated melanized fungi in the tropics interact with arboreal ants. It is noteworthy that the clade of domatia-associated Chaetothyriales is sister to a pair of species known to be epiphyllous (i.e. living on the surface of tree leaves). In addition, the closest relatives of these clades are the families Cyphellophoraceae and Phaeosaccardinulaceae, mostly composed of plant-associated species (Quan et al., 2020). Domatia species also occur in Cyphellophoraceae, but also in Herpotrichiellaceae and Trichomeriaceae which have many epiphyllous ones (Quan et al., 2020; Quan et al., 2023). The scraping of leaf surfaces by arboreal ants — most probably to collect epiphylls for larval feeding, as observed today (Davidson et al., 2003; Davidson et al., 2016) — may have provided many opportunities over time for epiphyllous Chaetothyriales and Capnodiales to adapt and colonize in the environment of newly emerging domatia, where their tolerance to different chemical compounds would give them an advantage over competitors. However, Baker et al. (2017) found a marked difference between the fungal community in the domatia of African *Vachellia drepanolobium* and the one on the surface of leaves of the same plants. A comprehensive study of the epiphyllous melanized fungi would probably substantially improve our understanding of the evolution of the ant-plant associated species.

Similarly, most carton-associated Chaetothyriales from the family Trichomeriaceae are embedded in a clade of epiphyllous species, and Capnodiales are known to be epiphyllous and colonizers of honeydew on leaf surfaces (Abdollahzadeh et al., 2020).

## 5 Specificity and transmission

### 5.1 Various degrees of specificity in ant-associated fungi

The degree of specificity between ants and fungi depends on the taxonomic level and differs between the fungi involved. At the species level, some fungal strains isolated from galleries made of carton walls or domatia are ubiquitous and not specific to either the ant or the plant species (e.g., Nepel et al., 2016), whereas other strains have been found to show a high degree of ant-host specificity (e.g., Ruiz-Gonzalez et al., 2011; Blatrix et al., 2013).

### 5.1.1 Examples for low specificity in ant-associated fungi

*Azteca brevis* ants living on *Tetrathylacium macrophyllum* build galleries that form paths with carton sidewalls and a roof, allowing them to move quickly and safe along branches and stems (= runway-galleries) (**Figures 1I-L**). Mayer and Voglmayr (2009) found a complex association of fungal species belonging to the Chaetothyriales order. Specifically, Nepel et al. (2014) found 62 different “Operative Taxonomic Units” (OTUs), the most common OTU was represented in 63% of the investigated trees. *Azteca brevis* does not seem to strongly select for a particular morphological type; this ant species cultivates and uses many different kinds of Chaetothyriales, suggesting a low specificity that could result from an environmental acquisition of fungal strains able to grow on such carton galleries (Mayer and Voglmayr, 2009).

The African ant-plant *Vachellia* (*Acacia*) *drepanolobium* is associated with three main obligate plant-ants: *Tetraponera penzigi*, *Crematogaster nigriceps* and *C. mimosae*. Baker et al. (2017) found that each of these ants is associated with a distinctive fungal community inside the domatia. Most of the fungi were plant pathogens or saprotrophs whose presence in a natural plant environment is quite plausible, suggesting that these ant-fungal associations are opportunistic. Chaetothyriales fungi were also not prominent in this association.

In the interaction between *Crematogaster borneensis* and *Macaranga bancana*, Hirose et al. (2013) found 15 OTUs, none of which were from the order Chaetothyriales. In contrast, Voglmayr et al. (2011) previously isolated a single representative of this fungal order in the same system, which belonged to the monophyletic clade of domatia inhabiting Chaetothyriales. These contrasting observations are likely the result of the use of different isolation methods to get fungal cultures: while Voglmayr et al. (2011) isolated the fungi directly from fungal “patches” of freshly opened domatia still inhabited by ant colonies, the method of Hirose et al. (2013) was rather unspecific, including a three week incubation step of the cut domatia before isolation of pure cultures from hyphae. Greenfield et al. (2021) identified fungal OTUs from the orders Chaetothyriales and Capnodiales, but also from other fungal orders in the interaction between *Philidris cordata* and *Myrmecodia beccarii*. In terms of the overall proportion of OTUs, Chaetothyriales were the dominant domatia inhabiting fungi. Within the pitcher leaves of *Dischidia major* occupied by *Philidris* sp. ants Blatrix et al. (2021) found nine species of Black Fungi belonging to Chaetothyriales (5) and Capnodiales (4), among which two have already been isolated from ant-plant mutualisms in Africa and South America. Many fungal associates are likely pantropical ant-plant associated species.

### 5.1.2 Examples for high specificity

High specificity in ant-plant-fungi interactions occurs in a wide diversity of systems. In these interactions, typically only a few strains of Chaetothyriales comprise the fungal mutualists, either in galleries built by the ants or in fungal patches within domatia.

Ants from the genus *Allomerus* build galleries along their host plant stems that are structurally similar to the ones built by *A. brevis* (Mayer et al., 2017) and used to ambush for prey (Dejean et al., 2005) (**Figures 1M-O**). In contrast to *A. brevis* ants, which have a guild of different Chaetothyriales in their galleries (Nepel et al., 2014) (see also 5.1.1), the three *Allomerus* species studied so far appear to be mainly associated with a single fungal species of Chaetothyriales, *Trimmatostroma cordae* (Ruiz-Gonzalez et al., 2011).

Ant species-specific fungal strains in domatia are known from the African ant-plant interactions *Petalomyrmex phylax* – *Leonardoxa africana* subsp. *africana*, *Tetraponera aethiops* – *Barteria fistulosa* (**Figures 2A, B**), *T. latifrons* – *B. dewevrei* and *Crematogaster margaritae* – *Keetia hispida* (Voglmayr et al., 2011; Blatrix et al., 2013; Kokolo et al., 2016). Blatrix et al. (2013) detected that 98% of the fungal taxa in these ant-plant interactions belong to the Chaetothyriales. Each ant-plant interaction was associated with a specific and dominant fungal taxon, indicating a high specificity and constancy in the composition of the fungal community. In one study site, the interactions involving *T. aethiops* – *B. fistulosa* and *T. latifrons* – *B. dewevrei* were preferentially associated with two sister OTUs of Chaetothyriales fungi, namely Y1 and Y9, respectively (Kokolo et al., 2016). In another site, these authors found that both fungal strains Y1 and Y9 were equally represented in the *T. aethiops* – *B. fistulosa* interaction.

Furthermore, in the Central American *Azteca* – *Cecropia* ant-plant association, Nepel et al. (2016) found a total of six fungal OTUs belonging to chaetothyrialean fungi when investigating the dark ant-made patches in the hollow stems of all *Cecropia* plants inhabited by *Azteca* ants (*A. alfari*, *A. coeruleipennis*, *A. constructor*, *A. xanthochroa*) (**Figures 2E-J**). One fungal OTU, OTU2, was preferred by *Azteca alfari* and was consistently found with this ant species. In contrast, the other three *Azteca* species were more frequently found with multiple OTUs often even in the same colony, and often OTUs different from the OTU associated with *A. alfari*. In addition, the finding that *A. alfari* colonies from three different geographic regions cultivated fungi of OTU2 while *A. constructor* predominantly cultivated fungi of OTU3, indicates a certain level of specificity at least for these two species (Nepel et al., 2016).

### 5.2 Mode of transmission

#### 5.2.1 Vertical transmission

The acquisition and eventual transmission of fungal partners across generations has not been investigated in detail thus far. Nevertheless, insights can be drawn from existing information. Fungi are absent from myrmecophytic plants that have not been colonized by ants (Defossez et al., 2009; Ruiz-Gonzalez et al., 2011; Mayer et al., 2018). But soon after the colonization by founding queens they are present (Mayer et al., 2018) which indicates that the inoculation comes *via* the ants, and not from the plant tissue. Vertical transmission of associated fungi (i.e., direct transmission from mother to daughter colony) is common in insect-fungus mutualisms (Biedermann and Vega, 2020). This mode of transmission is known from attine ants (Chapela et al., 1994; Mueller et al., 1998) and has also been suggested for *Lasius fuliginosus* ants which cultivate fungi on their nest walls (Schlick-Steiner et al., 2008). Such a transmission mode ensures that the right partners get involved in the next generation of mutualism. In ant-plant mutualisms, vertical transmission of the associated fungi is strongly suspected for the following reasons:

- The occurrence of a single fungal species (*Trimmatostroma cordae*) on the galleries of all three *Allomerus* spp. argues for a specific association between the *Allomerus* ants and the fungus, and thus towards host specificity (Ruiz-Gonzalez et al., 2011).
- Fungi from the so-called “domatia symbiont clade” of the Chaetothyriales have only been found in domatia of myrmecophytes inhabited by mutualistic ants and are not yet known from any other substrate (Mayer et al., 2018; Ruiz-González et al., 2019). Notably, there is no overlap with the fungi growing on the carton galleries of *Azteca brevis*, which contain a high species biodiversity of fungi from three different clades within the Chaetothyriales, but not from the so-called “domatia symbiont clade” (Mayer and Voglmayr, 2009; Voglmayr et al., 2011; Nepel et al., 2016).
- The presence of a single Chaetothyriales OTU in *Cecropia* trees inhabited by *Azteca alfari* in different geographical regions suggests mother to daughter transmission rather than a random contamination of alate queens from the environment (Nepel et al., 2016; Mayer et al., 2018). If multiple “domatia symbiont” OTUs occur in a single colony, this may be related to the cooperative multi-queen colony founding (pleometrosis) which has been observed in some ant-plant mutualisms (e.g. in *Triplaris* – *Pseudomyrmex* and *Cecropia* – *Azteca*; Perlman, 1992; Choe and Perlman, 1997; Sanchez, 2016; Mayer et al., 2018). Queens of the same ant species or with mixed species, each with its own infrabuccal pocket content, are together in a single domatium taking care of the initial patch and the first eggs (Mayer et al., 2018). However, colonies with >100 workers are usually single-queen colonies, and co-foundresses have been found cut into pieces within the patch material (Perlman, 1992; Choe and Perlman, 1997; Sanchez, 2016; Mayer et al., 2018). For *Cecropia*, it has also been observed that unless there is >1 queen on an internode, young plants usually have several internodes occupied by individual queens founding their own colony (Mayer et al., 2018). Once the dominant colony has conquered the entire host plant, a higher OTU diversity also occurs.
- The presence of hyphae and fungal spores in the infrabuccal pockets of alate queens point toward fungal dispersion between host myrmecophytes and inoculation of the domatia with fungal pellet material brought from the fungiculture of their mother colonies (Baker et al., 2017; Mayer et al., 2018). Mayer et al. (2018) were unable to generate DNA sequences from the infrabuccal pockets, so it was uncertain whether the hyphae and spores found are truly those that occur in the domatia fungal community. However, eggs and larval stages of nematodes of the order Rhabditidae were also detected in the infrabuccal pockets. but are never observed on the surface of the area where the ants chew the entrance hole to the hollow stem of their host plant (=prostoma) (Maschwitz et al., 2016; Mayer et al., 2018). Therefore, it is highly probable that the foundress queens take a piece of the fungal culture before leaving the nest for swarming. Baker et al. (2017) found that a substantial fraction of the fungal sequences from *V. drepanolobium* domatia was recovered from *T. penzigi* and *C. nigriceps* alates but infrabuccal pockets samples also contained fungi that were not found from domatium samples. This may reflect either variation among host plants not captured by the sampling design, or that alates acquire fungi from sources other than the domatia of their mother colony (Baker et al., 2017).

#### 5.2.2 Horizontal transmission

Some fungal strains isolated from galleries or domatia are ubiquitous and seem not to be specific to the ant species pointing towards an environmental acquisition (Hirose et al., 2013; Nepel et al., 2014; Nepel et al., 2016; Vasse et al., 2017; Greenfield et al., 2021). Ant workers can bring spores or hyphal fragments from the environment into the nest by collecting pieces of soil and various organic debris from the ground or the canopy as has been highlighted in other interactions (Abbott, 2002; Leroy et al., 2022). *De novo* acquisition of fungal species from the environment at each ant generation would explain the high number of genotypes and OTUs in domatia that do not cluster in the domatia symbiont clade (Voglmayr et al., 2011; Vasse et al., 2017; Greenfield et al., 2021). Nest wall carton and galleries that are exposed to the environment can be colonized by spores from fungi that can use the substrate and cope with the ants’ chemistry (see 6.4). For example, the community of Chaetothyriales fungi on the carton galleries built by *A. brevis* may be a subset of the fungal community found on the surface of the host plant (Nepel et al., 2014). It is likely that many of these strains are opportunistic and not mutualists, or even parasitic.

## 6 Where are we in the understanding of the roles of the associated fungi?

### 6.1 Reinforcement and stability of ant constructions

Fungi found on the nest walls of free-hanging nests, runway galleries or “carton” structures surrounding domatia entrances, or flocks of scale insects, increase the stability of nest walls and gallery structures through a dense network of interwoven and overlapping hyphae of thick-walled melanized Chaetothyriales and Capnodiales. While freshly built walls without fungi are unstable and easily disintegrate when touched, walls covered with the fungal network are very stable and particularly flexible when wet (Weißflog, 2001; Mayer and Voglmayr, 2009; Vogel, 2012). Rhizoids attach the hyphae to the host plant branches or stems, thus firmly connecting the ant nest walls to the substrate (Weißflog, 2001). This enhances the nest architecture and allows the development of unique defence and prey capture techniques, as documented in the associations between the two *Allomerus* species and *Hirtella physophora*, and *Tetrathylacium macrophyllum – Azteca brevis* (Dejean et al., 2005; Mayer and Voglmayr, 2009; Mayer et al., 2017). Furthermore, the fungal network quickly absorbs water, protecting the nest interior from being flooded during heavy rains common in the tropics, while also appearing to be drought tolerant (poikilohydric) and can sustain periods of low water availability (Weißflog, 2001; Mayer and Voglmayr, 2009; Vogel, 2012).

While building their runway galleries, *Allomerus* workers were observed gluing pellets from scraped epidermis and mesophyll of the inner domatia walls onto the gallery frame built from trichomes of the host plant (Ruiz-Gonzalez et al., 2011). Video recordings by Weißflog (2001) of Southeast Asian *Technomyrmex* sp. and *Monomorium* sp. during construction showed that the workers were continuously applying droplets of rectal fluid to the building material and to newly constructed parts. This suggests a purposeful substrate preparation to enhance fungal growth and increase the construction stability.

### 6.2 Food for the offspring

It has been suggested for a long time that the fungi found on ant-built structures and in domatia are consumed by the ants (Miehe, 1911; Bailey, 1920). However, experiments involving *Technomyrmex* workers kept with nest wall fungi (Weißflog, 2001) did not provide evidence of feeding on hyphal fragments. This is not surprising for nest wall fungi, which have thick, melanized walls, making them an unlikely digestible food source. In contrast, since domatia fungi have thin, hyaline walls (Voglmayr et al., 2011), and as fungi are nutritious and consumed as a food source by many insects (Biedermann and Vega, 2020), it is obvious to assume that they could also be consumed by plant-ants. Blatrix et al. (2012a) showed for three other ant-plant associations (*Petalomyrmex phylax* - *Leonardoxa africana*, *Tetraponera aethiops* - *Barteria fistulosa*, *Pseudomyrmex penetrator* - *Tachigali* sp.) that chaetothyrialean fungi are used as a food source for larvae, although not consumed daily. The extent to which myrmecophytic ants depend on fungi for food is unknown, but the major food sources may be from the host plant. The ants can feed directly, and even exclusively, on plant-derived food in the form of extrafloral nectar rich in carbohydrates, or food bodies rich in proteins (Heil et al., 1998), lipids (Fischer et al., 2002) or both (Folgarait and Davidson, 1995), or phyto-glycogen (Bischof et al., 2013). Most plant-ants also tend Hemiptera inside domatia for the carbohydrate-rich honeydew and for ‘meat’ as source of protein or lipids (Wheeler, 1942; Carroll and Janzen, 1973; Gullan, 1997; Dill et al., 2002). If the plant is not providing food sources, the diet is mainly based on prey. Fungi may be necessary as a food source, if they provide micronutrients unavailable from other sources, if the ant population grows faster than the food provided by the plant due to factors such as drought, lack of light or poor soil conditions, or if there are no coccids inside the domatia to supplement nitrogen-poor extrafloral nectar.

### 6.3 Recycling of macromolecules and indirect/direct transfer of N to the plant tissue

One potential function of the fungal patches appears to be the recycling and storage of nutrients. Ants feed the fungal mutualist with their faeces and provide hyphae to their larvae, suggesting a recycling role of the fungus (Blatrix et al., 2012a; Defossez et al., 2011). Additionally, the patch is enriched with nitrogen from its atmospheric nitrogen-fixing bacteria (Nepel et al., 2022), which further minimises the dependence on nitrogen from outside the ant-plant system. Nitrogen (N) – one the major elements for animal growth – is often in short supply for tropical arboreal ants (Tobin et al., 1995; Davidson et al., 2003). This also applies to phosphorous (P), which has been shown to play an important role in the ants’ ability to cope with thermal stress in the hot canopy (Kaspari et al., 2016). The recycling of macronutrients within the ant-plant system may be very important, especially for ants that feed on N-poor diets such as extrafloral nectar, plant secretions, or honeydew from scale insects.

Another function, only recently discovered, is the transfer of nutrients into the host plant tissue. Leroy et al. (2017) and Gegenbauer et al. (2023) identified hyphae inside stem tissues and revealed that the fungi actively transfer nitrogen into the plant tissues. In the orchid *Caularthron bilamellatum* it was demonstrated that hyphae assigned to Black Fungi (Chaetothyriales, Cladosporiales, Mycosphaerellales) rapidly transport nutrients from ant waste to a transition zone where it can be taken up and translocated to the vessels (Gegenbauer et al., 2012). Defossez et al. (2011) showed that part of the nitrogen introduced in *Petalomyrmex* - *Leonardoxa* was cycling in the system and there were reciprocal exchanges of nitrogen among the three partners. More detailed examination of the specialization in ant-plant interactions and nutritional fluxes among all partners might provide additional clues on the degree of specificity of fungal association.

### 6.4 Biofilter and nest hygiene

To protect their colony, ants regularly disinfect their nest and brood with a variety of antimicrobial substances (Tragust et al., 2013). Taken together, over 40 anatomically distinct exocrine glands are present on ants’ bodies and legs (Hölldobler and Wilson, 1990), which produce a huge variety of chemical compounds that ants use to organize the colony and protect the brood and adult nest members against pathogens (Attygalle and Morgan, 1984; Hölldobler and Wilson, 1990; Wang et al., 2015a). All organisms within ant nests are not only exposed to these chemicals, they are also exposed to plant-emitted substances. Plants themselves produce volatile organic compounds (VOCs) from various chemical classes, such as terpenoids, benzenoids and phenylpropanoids, as well as fatty acid-derived molecules or sulfides for communication with the organisms in their environment (Bouwmeester et al., 2019), in response to herbivory, and during normal plant growth, development and maintenance (Dani and Loreto, 2022). Some of the plant- and ant-emitted VOCs, such as benzothiazole, carbon disulphide, acetaldehyde, *p*-cymene, D-limonene (Hubert et al., 2008; Verza et al., 2011), are known to have insecticidal properties, and the accumulation of these chemicals could be deleterious to the vulnerable larval stage of the ants.

The concentration of VOCs was measured for the first time in the *Azteca*/*Cecropia* association. Surprisingly, the concentrations of most aldehydes, aromatic compounds, sulphur-containing compounds, and terpenes were, on average, higher in uninhabited domatia than inhabited ones. This was despite the fact that entrance holes of the inhabited domatia where the VOCs were measured were too small for efficient ventilation (Mayer et al., 2021). As several studies have demonstrated the ability of Chaetothyriales to degrade hydrocarbons (Isola et al., 2013; Prenafeta-Boldú et al., 2018; Baron et al., 2021), the authors suggest that the melanized fungi growing in the domatia may be partly responsible for the reduced VOC concentrations (Mayer et al., 2021). However, confirmatory experiments are still needed to clarify whether the VOCs are harmful to the larvae and whether Chaetothyriales in the domatia bind and degrade some of the VOCs. Another yet unexplored function of Black Fungi may be a possible contribution to domatia hygiene. In contrast to carton and other Chaetothyriales, domatia-associated Chaetothyriales show a remarkable increase of gene clusters related to secondary metabolism, such as modular polyketide synthases (type-I PKS) and non-ribosomal peptide synthetases (NRPSs) (Moreno et al., 2019). These are known to be involved in the synthesis of some of the most important antibiotics and anti-parasitics (Fischbach and Walsh, 2006; Süssmuth and Mainz, 2017), and their increase points to a specific function. However, confirmatory experiments are still needed to clarify whether Black Fungi in the domatia bind and degrade detrimental VOCs, and whether they produce antibiotics that act in the suppression of pathogens in the domatia.

## 7 Who are the fungi associated with?

Are the fungi more dependent on ants or plants species, or both? Globally, the presence of fungi in ant-plant interactions appears to depend more on the ants than on the plants. In *Azteca* – *Cecropia* associations, the identity of the associated fungi was not dependent on the host plant species but on the associated ant species (Nepel et al., 2016). Similarly, when *Cordia nodosa* is inhabited by *Azteca* sp. cf. *depilis*, a mutualist that does not build galleries on the plant like *A. octoarticulatus* does, no Chaetothyriales have been found (Ruiz-González et al., 2019). Instead, two fungal strains, from Pleosporales and from Trichosporales, have been identified in the associations involving *Azteca* sp. cf. *depilis* but not if inhabited by *Allomerus* spp. (Ruiz-González et al., 2019). Also in *Barteria dewevrei* we find this phenomenon: Chaetothyriales could only be isolated when inhabited by the mutualists *Tetraponera aethiops* or *T. latifrons*, but not if colonized by *Crematogaster* sp., a parasite (Kokolo et al., 2016). The host plant provides the environment, but the presence of Chaetothyriales as mutualistic partners seems to depend on the ant species.

## 8 Concluding thoughts

The progress of sequencing technologies opened the door to a world of less visible associates of ants. It allowed us to realize how frequent and widespread ecological relationships between Black Fungi and many different ant species are. Ant nests are important, previously overlooked habitats for Chaetothyriales and Capnodiales, the vast majority of which are still undescribed or only known as sequence.

In Chaetothyriales, the phylogenetic pattern (one monophyletic and well separated clade) and functional considerations (possible morphological adaptation and reduced gene functions) suggest that the domatia-associated species have coevolved with ants over a long period of time and have developed specific adaptations to this lifestyle, possibly representing an adaptive radiation. Further research, including a formal quantification of this trait and its mapping onto the phylogeny, would be a valuable aid for understanding the evolution of the ant-fungus mutualism in relation to the lifestyle of both partners.

There are, however, many open questions. Not all Chaetothyriales and Capnodiales detected may be mutualists, but to determine this requires a better understanding of the functional role of the associated fungi. Understanding the functional role also helps in understanding the evolutionary drivers which lead to a beneficial mutualism between ants and Chaetothyriales. Further, we assume that ant-associated Chaetothyriales growing in domatia have a long common history, but the origin of this fungi - ant nest mutualism is unclear. It would not be surprising to find that the ants have recruited the fungi from epiphyllous communities in their foraging environment, or that the fungi migrated into ant nests as a suitable niche and recruited the ants as their dispersal vector. A comprehensive study of the epiphyllous Chaetothyriales and Capnodiales as well as a higher number of genome analyses in ant-associated fungal species, would substantially improve our understanding of the evolution and functional role of the fungal species associated with ant and plant interactions.

Another still unexplored aspect is the defensive benefit of domatia and carton fungi for ants. While the antimicrobial activities of substances from bacterial symbionts in ants have been investigated (Seipke et al., 2012; Seipke et al., 2013; Hanshew et al., 2015; Fukuda et al., 2021), the role of fungi for protection against pathogens or otherwise detrimental fungi is yet to be considered. There are remarkable examples in other insects where the occurrence of *Penicillium* protects the larvae (Wang et al., 2015b). Though gene clusters in domatia-associated fungi point to a protective role, it is unknown whether and which substances are released and what their effect might be.

## Author contributions

VM wrote chapters 1, 2, 6 and 8. HV chapter 3. RB chapter 4. CL & JO chapters 5 and 7. All authors contributed to the article and approved the submitted version.
